# Long-Term Outcomes of Nasopharyngeal Carcinoma by Epstein–Barr Virus Status in the Chinese Population: A Multicenter Investigation

**DOI:** 10.3390/jcm12083005

**Published:** 2023-04-20

**Authors:** Yudi Xiong, Mengting Yuan, Zhigang Liu, Jing Huang, Jianping Bi, Guoliang Pi, Ying Li, Yanping Li, Hanping He, Vivek Verma, Suqing Tian, Guang Han

**Affiliations:** 1 Department of Radiation Oncology, Hubei Cancer Hospital, Tongji Medical College, Huazhong University of Science and Technology, Wuhan 430060, China; 2 Department of Oncology, The Fifth Affiliated Hospital, Sun Yat-sen University, Zhuhai 519000, China; 3 Cancer Center, Union Hospital, Tongji Medical College, Huazhong University of Science and Technology, Wuhan 430074, China; 4 Department of Radiation Oncology, University of Texas MD Anderson Cancer Center, Houston, TX 77030, USA; 5 Department of Radiation Oncology, Peking University Third Hospital, Peking University, Beijing 100191, China

**Keywords:** nasopharyngeal carcinoma (NPC), Epstein–Barr virus (EBV), prognosis

## Abstract

**Background:** Because the vast majority of nasopharyngeal carcinoma (NPC) in Chinese patients is a direct result of Epstein–Barr virus (EBV) infection, there is a dearth of data for EBV-negative patients in this population. This multicenter study sought to examine the clinical characteristics of EBV-negative patients and compare long-term outcomes with a propensity-matched (1:1.5) EBV-positive cohort. **Methods:** NPC patients with known EBV status from four hospitals were collated (2013–2021). A logistic regression model was conducted to evaluate the relationship between patient characteristics and EBV status. The Kaplan–Meier method and Cox regression analysis were used to analyze survival data. **Results:** This study analyzed 48 (40%) EBV-negative and 72 (60%) EBV-positive patients. The median follow-up time was 63.5 months. Most EBV-negative NPC patients (77.1%) were diagnosed in advanced stages with a higher rate (87.5%) of positive lymph node disease, and no significant prognostic factors were discerned in this subpopulation. The EBV-negative disease was more associated with the keratinizing subtype (18.8% vs. 1.4%, *p* < 0.05). Compared to EBV-negative NPC patients, EBV-positive NPC patients were more likely to develop a local recurrence (9.7% vs. 0%, *p* = 0.026). There was no statistical difference in mortality (EBV-negative vs. EBV- positive, 8.3% vs. 4.2%, *p* = 0.34) during the follow-up period. Although the median PFS and median OS were not reached, the 3-year PFS rate was 68.8% vs. 70.8% (EBV-negative vs. EBV-positive, *p* = 0.06), the 3-year OS rate was 70.8% vs. 76.4% (EBV-negative vs. EBV-positive, *p* = 0.464), the 5-year PFS rate was 56.3% vs. 50% (EBV-negative vs. EBV-positive, *p* = 0.451), and the 5-year OS rate was 56.3% vs. 58.3% (EBV-negative vs. EBV-positive, *p* = 0.051), respectively. These data show that EBV-positive NPC patients seem to have a tendency to gain better survival compared with EBV-negative NPC patients. **Conclusions:** Most of the EBV-negative patients were in the middle and late stages at the time of diagnosis and were more associated with the keratinizing subtype. EBV status may be associated with prognosis in NPC. EBV positivity seems to be associated with better survival in NPC patients. Still, due to the small cohort of patients and the short observation period for a number of patients, further work is required to corroborate these conclusions.

## 1. Background

Nasopharyngeal carcinoma (NPC) can be divided into three pathological subtypes: keratinizing squamous, non-keratinizing (including differentiated and undifferentiated), and basaloid squamous. Undifferentiated non-keratinizing NPC is the most common pathological classification of NPC and is associated with Epstein–Barr virus (EBV) infection, accounting for 95% of NPC cases in southern China [1,2,3].

EBV infects the host cells by expressing EBV-encoded transforming proteins and noncoding RNAs to alter multiple cellular pathways, promote cell proliferation, regulate the host microenvironment, and thereby, promote the clonal expansion of EBV-infected preinvasive nasopharyngeal epithelial cells [4]. The detection of EBV-encoded small RNA (EBER) in the nuclei of tumor cells by in situ hybridization (ISH) was widely used in NPC, and NPC patients were classified as EBV (+) or EBV (−) based on these results.

In previous studies, EBER positivity was associated with both improved overall survival (OS) and disease-free survival (DFS) [5]. EBV-negative NPC was correlated with worse overall survival (OS) [6]. Since most NPC cases in the Chinese population were EBV-positive, there was a lack of research on EBV-negative patients in China, including a lack of larger-scale studies on the clinical characteristics of EBV-negative patients.

In order to further study the clinical characteristics of EBV-negative patients and investigate the correlation between EBV infection status and prognosis in this population, we combined data from four centers in China to analyze the general clinical characteristics of EBV-negative patients and compared outcomes with a matched EBV-positive NPC cohort after long-term follow-up.

## 2. Materials and Methods

NPC patients were classified as EBV (+) or EBV (−) based on the detection of EBV-encoded small RNA (EBER) in the nuclei of tumor cells by in situ hybridization (ISH). We collated all EBV-negative NPC patients from four hospitals (Hubei Cancer Hospital of Huazhong University of Science and Technology, Tongji Hospital of Huazhong University of Science and Technology, The Fifth Affiliated Hospital of Sun Yat-Sen University and Peking University Third Hospital) from 2013 to 2021.

Baseline/demographic and treatment-related characteristics were collected for each patient as given in their medical record. The outcomes analyzed herein were progression-free survival (PFS), which was defined as the time from cancer diagnosis to the time of tumor progression (local, regional, or distant) or death from any cause, and OS, which was defined as the time from cancer diagnosis to the time of death from any cause or was censored at the last follow-up.

To perform a comparative analysis between EBV-positive and EBV-negative patients, a propensity score matching analysis was used to reduce the effect of treatment selection bias and simulate the effects of randomization. EBV-positive NPC patients treated in the same time period as above were selected according to a 1:1.5 pairing with EBER-negative NPC patients based on several known prognostic factors, including the Eastern Cooperative Oncology Group performance status (ECOG PS), sex, age, stage, and type of therapy.

Descriptive statistics were used to compare the clinical characteristics of EBV-positive and EBV-negative NPC patients. The categorical variables (frequency and proportion) were analyzed by chi-square tests. Logistic regression analysis was applied to analyze the associations between the EBV tumor status and clinicopathologic factors. Kaplan–Meier analyses were performed to generate survival curves, and the log-rank test was applied for a statistical comparison thereof. Comparative risk factors for PFS and OS were identified by univariate and multivariate analyses using Cox regression models. All statistical analyses were conducted with SPSS version 23.0 (IBM Corp., Armonk, NY, USA). All statistical tests were two-sided, and a *p*-value < 0.05 was considered statistically significant.

## 3. Results

A total of 120 NPC patients with a known EBV status were included in this study, including 48 (40%) EBV-negative patients and 72 (60%) matched EBV-positive patients. The median follow-up period for the entire study population was 63.5 months (6–96 months).

The clinicopathological features of EBV-negative NPC patients are shown in Table 1. The median age at diagnosis was 52 (range: 21–75) years, and 32 patients (66.7%) were male. Thirty-nine cases (81.3%) had the nonkeratinizing pathological subtype. The TNM stage distribution was as follows: Stage I (*n* = 1), II (*n* = 10), III (*n* = 24), and IV nonmetastatic (*n* = 13). Tumor stages were distributed as follows: T1 (*n* = 9), T2 (*n* = 15), T3 (*n* = 16), and T4 (*n* = 8). Nodal involvement was as follows: N0 (*n* = 6), N1 (*n* = 17), N2 (*n* = 19), and N3 (*n* = 6). Regarding treatment, almost all patients received concurrent chemoradiotherapy with or without induction chemotherapy or targeted therapy/immunotherapy. Seven (14.6%) of the 48 patients recurred, all of which were distant metastases (bone or lung), four (8.3%) of whom died.

Table 2 shows a comparison between EBV-negative NPC and the matched EBV-positive cohort. The former was more associated with the keratinizing subtype (18.8% vs. 1.4%, *p* < 0.05). Compared to EBV-negative NPC patients, EBV-positive NPC patients were more likely to develop a local recurrence (9.7% vs. 0%, *p* = 0.026). There was no statistical difference in mortality (8.3% vs. 4.2%, *p* = 0.34) during follow-up. There were no significant differences between EBV-negative NPC and EBV-positive NPC in any other characteristics.

A logistic regression model was then constructed to evaluate the relationship between patient characteristics and EBV status. As shown in Table 3, the NPC subtype (OR = 15.142, 95% CI: 1.741–131.695, *p* = 0.014) was associated with higher odds of having an EBV-negative status. There was no significant association between EBV status and age, sex, life history (smoking, alcohol), or TNM stage.

**Figure 1:**
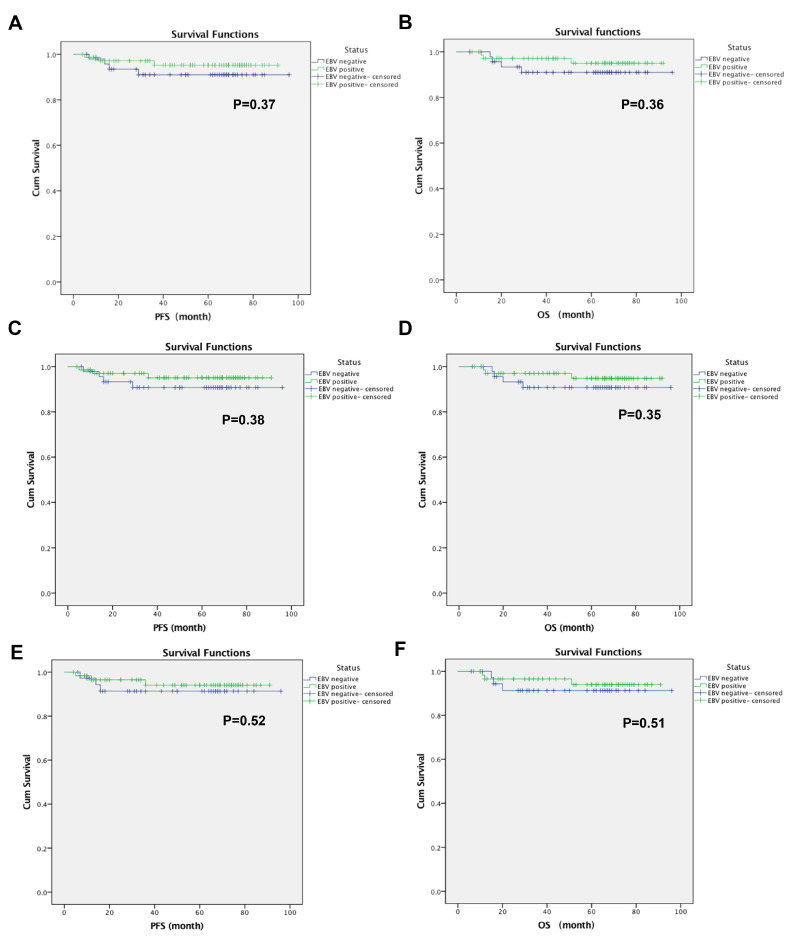
Impact of EBV status on survival of NPC patients. K–M plots of survival are shown for: (**A**) Cum survival of PFS, (**B**) Cum survival of OS in all stages of NPC patients; (**C**) Cum survival of PFS, (**D**) Cum survival of OS in stage II-IV NPC patients; (**E**) Cum survival of PFS, (**F**) Cum survival of OS in stage III-IV NPC patients. (*y*-axis: cum survival percentage (%)). EBV: Epstein–Barr virus; NPC: nasopharyngeal carcinoma; PFS: progression-free survival; OS: overall survival.

As shown in Table 1, the PFS and OS of the EBV-negative NPC patients had no significant correlation with age, sex, NPC subtype, social history (smoking, alcohol), and TNM stage by Cox regression models. Kaplan–Meier estimates suggested that EBV-positive NPC patients may have better survival than EBV-negative NPC patients, although the median PFS and median OS were not reached (Figure 1). The 3-year PFS rate was 68.8% vs. 70.8% (EBV-negative vs. EBV-positive, *p* = 0.06), and the 3-year OS rate was 70.8% vs. 76.4% (EBV-negative vs. EBV-positive, *p* = 0.464), the 5-year PFS rate was 56.3% vs. 50% (EBV-negative vs. EBV-positive, *p* = 0.451), and the 5-year OS rate was 56.3% vs. 58.3% (EBV-negative vs. EBV-positive, *p* = 0.051), respectively. As shown in Figure 1, there was no significant difference in the cumulative PFS between EBV-positive NPC and EBV-negative NPC (*p* = 0.37). There was also no significant difference in the cumulative OS between EBV-positive NPC and EBV-negative NPC (*p* = 0.36). In the subgroup analysis, there were no significant differences in PFS or OS between EBV-positive NPC and EBV-negative NPC in stage II-IV or III-IV NPC patients.

## 4. Discussion

Owing to its rarity in the Chinese population, little is known about the clinical characteristics of EBV-negative patients and the differences in prognosis between EBV-positive and EBV-negative NPCs. In this multicenter study, we found that the keratinizing NPC subtype was more common in EBV-negative NPC than in EBV-positive NPC and that EBV-positive NPC patients were more likely to develop a local recurrence (9.7% vs. 0%, *p* = 0.026).

Other reports of EBV-negative NPC in Chinese populations remain scarce. In one investigation, the mean OS of EBV-positive NPC patients was 57 months, and the mean DFS was 49 months; in the EBV-negative cohort, the mean OS was 43 months, and the mean DFS was 36 months [5]. Another publication suggested no significant difference in survival between EBV-negative and EBV-positive NPC patients [7]. Our study also did not discern significant differences in survival between EBV-negative and EBV-positive NPC patients but found that EBV-positive NPC patients had higher local recurrence rates. This seems to imply the need to strengthen local treatment in EBV-positive NPC patients and potentially systemic treatment in EBV-negative NPC patients.

Under these conditions, the treatment mode and efficacy of EBV-negative NPC were worth exploring. Induction chemotherapy followed by chemoradiotherapy has become the main treatment method and contributed to improvements in treatment outcomes [8]. The five-year OS rate was 74–88% [9,10,11]. However, the effect of radiotherapy alone was not inferior to concurrent chemoradiotherapy in patients with low-risk nasopharyngeal carcinoma [12]. In an international multicenter retrospective study [13], patients with NPC diagnosed between 2004 and 2017 in 36 hospitals in 11 countries were analyzed. The treatments that these patients received were divided into non-intensive treatment (NIT), including simple two-dimensional radiotherapy (RT), three-dimensional conformal radiotherapy (3D-CRT) or intensity-modulated radiation therapy (IMRT), and intensive treatment (IT), including concurrent chemoradiotherapy (CCRT) combined with induction or adjuvant chemotherapy. The five-year OS and DFS results of EBER (+) and EBER (−) after different intensities of treatment suggested that the OS of EBER (+) and EBER (−) NPC patients did not differ by treatment type. DFS was higher in EBER (+) NPC patients treated with IT than in EBER (−) NPC patients, whereas treatment type had no significant effect on DFS in EBER (−) NPC patients [13]. This study also showed that advanced stages and age (>65 years) were important factors affecting the prognosis of patients with EBER (+) NPC, while age (>65 years) was an important factor affecting the prognosis of patients with EBER (−) NPC. However, due to insufficient sample sizes, we did not observe the same results.

In addition, we explored the association between patient characteristics and EBV status. The results showed that the NPC subtype was closely related to EBV status. The keratinizing NPC subtype was more common in EBV-negative NPC than in EBV-positive NPC. Compared with EBV-negative patients, most NPC patients had nonkeratinizing tissue, which was more common in EBER (+) tumors (91% vs. 70%) [13]. Our study also confirmed this finding (98.6% vs. 81.3%). This may be related to EBV in the process of tumorigenesis; EBV infection was present in almost all undifferentiated NPCs and almost every NPC cell [14].

To our knowledge, this is the first multicenter retrospective study to describe the clinical features of EBV-negative NPC patients in the Chinese population. However, there are several limitations to this work. First, the retrospective nature and small sample sizes limit statistical robustness. Second, the manner of EBV testing (assay or technique) may limit reproducibility. Third, propensity matching is not meant to substitute for randomized data. Lastly, these data are not meant to identify the optimal treatment regimen or sequencing of therapies for EBV-negative NPC.

## 5. Conclusions

In conclusion, owing to the rarity in the Chinese population, little is known about the clinical characteristics of EBV-negative patients and the differences in prognosis between EBV-positive and EBV-negative NPCs. In this multicenter study, we found that the keratinizing NPC subtype was more common in EBV-negative NPC than in EBV-positive NPC and that EBV-positive NPC patients were more likely to develop a local recurrence (9.7% vs. 0%, *p* = 0.026). This may imply the need to strengthen local treatment in EBV-positive NPC patients and potentially the same for systemic treatment in EBV-negative NPC patients. Whether EGFR-targeted therapy (cetuximab or nimotuzumab) or immunotherapy (PD-1/L1) has differential effects dependent on EBV status requires further investigation.

## Figures and Tables

**Table 1:** Clinicopathological features of EBV-negative NPC patients.

Characteristics	EBV Negative N = 48	PFS	OS
Age	21–75 (52 ± 11)	*p* = 0.395	*p* = 0.403
ECOG PS (0–1)	48 (100%)		
Sex		*p* = 0.663	*p* = 0.664
Male	32 (66.7%)		
Female	16 (33.3%)		
NPC subtype		*p* = 0.073	*p* = 0.081
Keratinizing	9 (18.8%)		
Nonkeratinizing	39 (81.3%)		
Smoking		*p* = 0.663	*p* = 0.664
Yes	32 (66.7%)		
No	16 (33.3%)		
Alcohol		*p* = 0.663	*p* = 0.664
Yes	32 (66.7%)		
No	16 (33.3%)		
AJCC 8th stage		*p* = 0.103	*p* = 0.143
I	1 (2.1%)		
II	10 (20.8%)		
III	24 (50%)		
IV	13 (27.1%)		
Tumor stage		*p* = 0.157	*p* = 0.135
T1	9 (18.8%)		
T2	15 (31.3%)		
T3	16 (33.3%)		
T4	8 (16.7%)		
Nodal stage		*p* = 0.694	*p* = 0.848
N0	6 (12.5%)		
N1	17 (35.4%)		
N2	19 (39.6%)		
N3	6 (12.5%)		
Metastatic stage			
M0	48 (100%)		
M1	0 (0%)		
Therapy			
Radiotherapy	0 (0%)		
Chemotherapy	0 (0%)		
Radiochemotherapy	48 (100%)		
Other	0 (0%)		
Recurrence/progression			
Distant metastasis	7 (14.6%)		
Local recurrence	0 (0%)		
Follow-up			
Died	4 (8.3%)		
Surviving	44 (91.7%)		

EBV: Epstein–Barr virus; NPC: nasopharyngeal carcinoma; PFS: progression-free survival; OS: overall survival; ECOG PS: Eastern Cooperative Oncology Group performance status.

**Table 2:** Clinicopathological features of NPC patients according to EBV status.

Characteristics	Total N = 120 (100%)	EBV Negative N = 48 (40%)	EBV Positive N = 72 (60%)	*p* Value
Age	Mean 51 (SD 10)	Mean 52 (SD 11)	Mean 50 (SD 10)	*p* = 0.322
ECOG PS (0–1)	120 (100%)	48 (100%)	72 (100%)	
Sex				*p* = 0.533
Male	82 (68.3%)	32 (66.7%)	50 (69.4%)	
Female	38 (31.7%)	16 (33.3%)	22 (30.6%)	
NPC subtype				*p* < 0.05
Keratinizing	10 (8.3%)	9 (18.8%)	1 (1.4%)	
Nonkeratinizing	110 (91.7%)	39 (81.3%)	71 (98.6%)	
Smoking				*p* = 0.533
Yes	82 (68.3%)	32 (66.7%)	50 (69.4%)	
No	38 (31.7%)	16 (33.3%)	22 (30.6%)	
Alcohol				*p* = 0.533
Yes	82 (68.3%)	32 (66.7%)	50 (69.4%)	
No	38 (31.7%)	16 (33.3%)	22 (30.6%)	
AJCC 8th stage				*p* = 0.645
I	3 (2.5%)	1 (2.1%)	2 (2.8%)	
II	20 (16.7%)	10 (20.8%)	10 (13.9%)	
III	60 (50%)	24 (50%)	36 (50%)	
IV (IVA)	37 (30.8%)	13 (27.1%)	24 (33.3%)	
Tumor stage				*p* = 0.240
T1	16 (13.3%)	9 (18.8%)	7 (9.7%)	
T2	38 (31.7%)	15 (31.3%)	23 (31.9%)	
T3	45 (37.5%)	16 (33.3%)	29 (40.3%)	
T4	21 (17.5%)	8 (16.7%)	13 (18.1%)	
Nodal stage				*p* = 0.345
N0	11 (9.2%)	6 (12.5%)	5 (6.9%)	
N1	39 (32.5%)	17 (35.4%)	22 (30.6%)	
N2	53 (44.2%)	19 (39.6%)	34 (47.2%)	
N3	17 (14.2%)	6 (12.5%)	11 (15.3%)	
Metastatic stage				*p* = 0.101
M0	120 (100%)	48 (100%)	72 (100%)	
M1	0 (0%)	0 (0%)	0 (0%)	
Therapy				
Radiotherapy	0 (0%)	0 (0%)	0 (0%)	
Chemotherapy	0 (0%)	0 (0%)	0 (0%)	
Radiochemotherapy	120 (100%)	48 (100%)	72 (100%)	
Other	0 (0%)	0 (0%)	0 (0%)	
Recurrence/progression	20 (16.7%)	7 (14.6%)	13 (18.1%)	*p* = 0.617
Distant metastasis	13 (10.8%)	7 (14.6%)	6 (8.3%)	*p* = 0.281
Local recurrence	7 (5.8%)	0 (0%)	7 (9.7%)	*p* = 0.026
Follow-up				*p* = 0.340
Died	7 (5.8%)	4 (8.3%)	3 (4.2%)	
Surviving	113 (94.2%)	44 (91.7%)	69 (95.8%)	

EBV: Epstein–Barr virus; NPC: nasopharyngeal carcinoma; ECOG PS: Eastern Cooperative Oncology Group performance status.

**Table 3:** Associations between patient characteristics and EBV status.

Characteristics	Sig.	OR	95% CI for OR	
			Lower	Upper
Sex	0.463	0.726	0.309	1.707
Age	0.650	0.991	0.951	1.032
Subtype	0.014	15.142	1.741	131.695
TNM	0.730	0.854	0.348	2.094
T	0.366	1.298	0.737	2.286
N	0.350	1.387	0.698	2.757
M	1.000	804,737,392.939	0.000	

EBV: Epstein–Barr virus; OR: odds ratio.

## Data Availability

Data are available on request due to privacy/ethical restrictions. Data can be obtained by contacting the corresponding author’s email (hg7913@hotmail.com) and the first author’s email (ydxiong_whu@163.com).

## Abbreviations

NPC	nasopharyngeal carcinoma
EBV	Epstein–Barr virus
EBER	EBV-encoded small RNA
ISH	in situ hybridization
OS	overall survival
DFS	disease-free survival
PFS	progression-free survival
ECOG PS	Eastern Cooperative Oncology Group performance status
OR	odds ratio
